# Incidence, Clinical Features, and Outcomes of Transient Tachypnea of the Newborn at a Tertiary Care Center in Western India

**DOI:** 10.7759/cureus.23939

**Published:** 2022-04-07

**Authors:** Sanajay Chavan, Sudhir D Malwade, Soni Kumari, Balakrushna P Garud, Sharad Agarkhedkar

**Affiliations:** 1 Paediatrics, Dr. D. Y. Patil Medical College, Hospital & Research Centre, Pune, IND; 2 Neonatology, Dr. D. Y. Patil Medical College, Hospital & Research Centre, Pune, IND

**Keywords:** newborn distress, respiratory distress in newborn, transient tachypnea of newborn, ttn, ttnb

## Abstract

Background

Transient tachypnea of the newborn (TTN) is a self-limiting, benign condition leading to respiratory distress shortly after birth. It is among the leading cause of respiratory distress in term and late preterm neonates. The disease is transient and resolves by three to four days in most neonates.

Objective

The objective of this study was to study the incidence of TTN, its clinical features, predictors of outcomes and duration of hospital stay in these neonates suffering from it.

Methods

This was a prospective study done at a tertiary care center carried out between August 2019 to July 2021. The study subjects were late pre-term (34 to 36 weeks of gestation) and term neonates with respiratory distress who were admitted to the neonatal intensive care unit (NICU). The diagnosis was based on clinical features, radiological features, and clinical course in NICU.

Results

The total number of cases with TTN was 74. The incidence of TTN was 16 per 1000 live births. 63.5% were male, 75.7% were term births, 70.3% were born via lower section cesarean section (LSCS), and 66.2% were normal birth weight (≥2.5 kg) infants. A high incidence of TTN was found in late pre-term babies, babies born via LSCS, and male sex. None of the neonates required ventilatory support, either noninvasive or invasive.

Conclusion

Delivery by LSCS and male sex were risk factors for the development of TTN. The distress in TTN is usually mild to moderate, and in most cases, oxygen supplementation suffices. Higher Downes’ score at presentation, low birth weight, preterm, and delivery by LSCS were found to be predictors for a longer duration of distress and thus the longer duration of NICU stay. Although severe complications for TTN have been reported in the literature, they are rare. Careful observation can decrease not only a lot of unnecessary investigations but also allow clinicians at secondary and primary centers to better care for neonates with TTN.

## Introduction

Transient tachypnea of the newborn (TTN) is usually a self-limited benign condition seen commonly in term and late preterm babies and most recover by 72 hours after birth. It is due to delayed clearance of fluids from the lungs after birth. The incidence ranges from 4.0-5.7 per 1000 live births in high-income countries to 13 per 1000 live birth in India.

Management is supportive and most neonates require supplemental oxygen with fraction of inspired oxygen (FiO2) no more than 0.40, if a higher oxygen concentration (>60%) is needed ventilatory support (continuous positive airway pressure [CPAP]) should be given [1]. Though transient in nature the initial features of TTN are similar to those of respiratory distress syndrome, pneumonia, and persistent pulmonary hypertension (PPHN), which often leads to unnecessary antibiotic therapy, imaging, and laboratory workup. This becomes important in developing countries where the economic burden on patients and scarce healthcare resources are major issues. Many newborns with TTN are needlessly referred to higher centers, adding to the costs and strain on already stretched thin health services. In rare cases, TTN may worsen into prolonged tachypnea (lasting more than five to six days) which may result in respiratory failure (characterized by a triad of hypoxia, respiratory fatigue, and acidosis) [2]. TTN may deteriorate into PPHN, this is known as 'malignant TTN', but the incidence has been rare [3]. The economic and familial implications of respiratory distress in neonates needing admission to the neonatal intensive care unit (NICU) may be far-fetching and in resource-limited countries may lead to economic burden on families. Therefore the primary aim of this study is to assess the incidence, clinical features, and outcomes of TTN, and the factors that might affect the outcomes and duration of hospital stay in neonates with TTN. The data generated from this study might help in counseling the parents and determining their health priorities.

## Materials and methods

**Figure 1:**
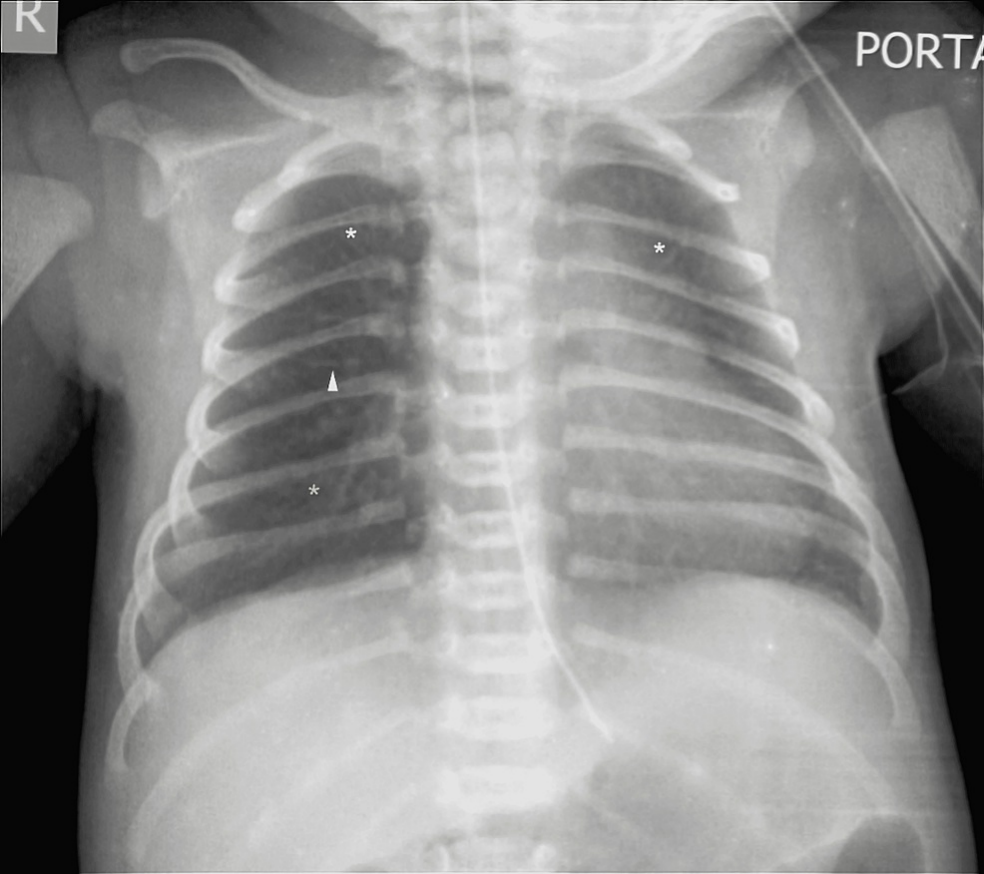
Chest radiograph in transient tachypnea of the newborn The radiograph shows typical features of TTN. Straightened ribs and increased intercostal space due to mild hyperinflation lungs. streaky infiltrates (white asterisks), fluid in horizontal lung fissures (white arrow), perihilar streaking (Sunburst)

This was a prospective, cross-sectional observational study carried out between August 2019 and July 2021. Due approval was taken from the Institutional Ethics Sub-committee at Dr. D. Y. Patil Medical College, Hospital and Research Centre, Pune (approval number: IESC/160/2019). All the newborns who were delivered at this hospital and admitted to our NICU for respiratory distress were followed up. Neonates with delayed transition were excluded. Delayed transition is transient respiratory distress that resolves within a few hours (six hours) of birth [2]. Patients with pneumonia, congenital heart disease, perinatal asphyxia, congenital malformations, and early-onset sepsis were also excluded. Detailed perinatal and post-natal history was taken to rule out any other causes of distress. Chest X-rays, complete blood count (CBC), and a sepsis screen were organized. Diagnosis of TTN was that of exclusion and based on clinical and radiological features. Chest X-ray features (Figure 1) include diffuse streaks of perihilar interstitial opacities (sunburst) and fluid in the interlobar fissures. This is due to the retained lung fluids which engorge the lymphatics and the capillaries. Some degree of hyperinflation with fluid in costophrenic angles may be present. Hyperinflation causes the widening of intercostal spaces and straightening of the ribs. Mild cardiomegaly might be present. Rapid clearing of the successive chest X-rays within 48-72 hours is a hallmark of TTN [4,5].

Oxygen supplementation was given via hood or nasal prongs. The oxygen saturation of the neonates was constantly monitored and the response to oxygen supplementation was evaluated. Invasive and non-invasive continuous positive airway pressure (CPAP) support were reserved for those neonates who had severe distress, or those who required higher concentrations of oxygen (>60%) to maintain their saturation. Oral feeds were started by spoon or paladai for babies with mild respiratory distress (respiratory rate 60-80/minute). Oral feeds were avoided in those with moderate to severe distress or those who had a significant increase in the work of breathing. These neonates were administered gavage feeding along with intravenous fluids which included 10% dextrose, electrolytes, and multivitamins. As tachypnea improved (respiratory rate: <80/min), babies were gradually weaned off oxygen and oral feeds were started. The neonates were discharged from NICU 12 hours after their tachypnea had resolved. Other causes of distress were actively evaluated if there was no improvement in symptoms on oxygen supplementation, if they required a very high concentration of oxygen, if the distress did not resolve beyond 72 hours, or if the X-ray did not improve after 48 hours. The data was recorded in MS Office Excel tables (Microsoft, Redmond, WA, USA) and analyzed using IBM SPSS 28.0.0 statistical software suite (IBM Corp., Armonk, NY, USA). Groups were evaluated using Student’s T-test, Man-Whitney U tests, Kruskal-Wallis test, and Rank sum test. The Chi-Square test was used for the comparison of proportions. Bivariate analysis was used to compare two numerical parameters. Statistical significance was determined as p < 0.05.

## Results

The total number of live births at our institution was 4562. There were 1289 NICU admissions during this period. Of these, 987 neonates were ≥34 weeks of gestation. A total of 189 neonates with respiratory distress fulfilled the inclusion criteria, these cases were followed. Out of 189 cases, 74 cases were of TTN, 70 cases of meconium aspiration syndrome (MAS), 15 cases of persistent pulmonary hypertension (PPHN), and 30 cases of respiratory distress syndrome (RDS). TTN cases were further analyzed. The demographic data of the neonates diagnosed with TTN is summarized in Table 1. 

**Table 1:** Demographic profile of the study population (n=74) SD – Standard deviation, LSCS – Lower segment cesarean section, VD – Vaginal delivery, GDM – Gestational diabetes mellitus, AGA – Appropriate for gestational age, SGA – Small for gestational age, LGA – Large for gestational age, NBW – Normal birth weight, LBW – Low birth weight, hrs. – Hours, Kg – Kilograms

Demographic variables	Subcategories	value
Maternal age	Mean age ± SD	23.99 ± 2 yrs.
Parity	Multigravida	38 (51.4%)
	Primigravida	36 (48.6%)
Maternal comorbidities	Hypothyroid	1(1.4%)
	GDM	2 (2.7%)
	Pre-eclampsia	2 (2.7%)
	No comorbidities	69 (93.2%)
Mode of delivery	LSCS	52 (70.3%)
	NVD	22 (29.7%)
Gender	Female	27 (36.5%)
	Male	47(63.5%)
Birth weight	Mean Birth Weight ± SD	2.6327 ± 0.44 Kg
	LBW	25(33.8%)
	NBW	49 (66.2%)
Gestational age	Mean Gestational age ± SD	37.45 ± 1.19 weeks
	Late preterm	18 (24.3%)
	Term	56 (75.7%)
	AGA	68 (91.9%)
	LGA	2 (2.7%)
	SGA	4 (5.4%)
Duration of distress	Mean Duration of Distress ± SD	30.55 ± 13.57 hrs.
	1-24 hrs.	41.0 (55.4%)
	25-48 hrs.	29.0 (39.2%)
	49-72 hrs.	4.0 (5.4%)
Downes’ score	1-4	57 (77%)
	5-6	17 (23%)
	>6	0

The incidence of TTN was found to be 16 per 1000 live births. 5.7% of all patients admitted to the NICU were TTN. Seventy percent (n=52) of newborns were delivered via LSCS, while 30% (n=22) were delivered by vaginal delivery. No difference was observed between both groups regarding gestational age, maturity, or birth weight. A significantly longer duration of distress was seen in neonates born via LSCS as compared to those born via vaginal delivery. 93.2% (69/74) mothers had no comorbidities, while two suffered from preeclampsia, two from gestational diabetes mellitus, and one from hypothyroidism. All the mothers with comorbidities had delivered via LSCS. No significant difference was observed among them concerning the duration of tachypnea. In this study, 33.8% of the neonates were LBW (<2.5 Kg). The rest of the neonates were normal birth weight (NBW). None of the neonate had macrosomia (> 4 Kg). The tachypnea had resolved in a more than half (55.4%) of the patients by 24 hours, and all the patients had successfully recovered by 72 hours. Birth weight and gestational age were independently correlated with the duration of tachypnea/distress. Duration of distress was inversely proportional to both birth weight (p<0.01) and gestational age (p=0.01). Duration of tachypnea was significantly longer in preterms, low birth weight newborns (LBW), and whose Downes' score was 5-6 (Table 2).

**Table 2:** Association of duration of distress with maternal and neonatal variables LSCS – Lower segment caesarean section, GDM – Gestational diabetes mellitus, AGA – Appropriate for gestational age, SGA – Small for gestational age, LGA – Large for gestational age, NBW – Normal birth weight, LBW – Low birth weight

Maternal and neonatal variables		Mean duration (hours)	p value
Birth weight	LBW	38.9	0.048
	NBW	26.3	
Gestational age	Late Preterm (34-36 weeks)	37.7	0.014
	Term (≥37 weeks)	28.3	
	AGA	29.5	0.267
	LGA	48.0	
	SGA	39.0	
Mode of delivery	LSCS	32.6	0.048
	Vaginal delivery	26.2	
Gender	Male	29.0	0.316
	Female	31.4	
Maternal comorbidities	Hypothyroid	36.0	0.441
	GDM	30.0s	
	Pre-eclampsia	42.0	
	No comorbidities	30.2	
Downe’s score	1-4	27.6	0.003
	5-6	40.2	

We divided the neonates into two groups: those whose tachypnea had resolved within 24 hours and those who had tachypnea lasting greater than 24 hours. We found significant differences among the groups based on the mode of delivery, birth weight, and Downes' score. Newborns born vaginally, NBW infants, and those with lower Downes' score (1-4) on presentation had more chances of recovering within 24 hours (Table 3).

**Table 3:** Comparison of characteristics of newborns who recovered within 24 hours to those who recovered >24 hours. LSCS – Lower segment caesarean section, NVD – Natural vaginal delivery, GDM – Gestational diabetes mellitus, NBW – Normal birth weight, LBW – Low birth weight

Maternal and Neonatal variables		No. of cases		P values
		≤24 hrs	>24 hrs.	
Parity	Multigravida	21 (55.3%)	17 (44.7%)	0.980
	Primigravida	20 (55.6%)	16 (44.4%)	
Mode of Delivery	LSCS	23 (44.2%)	29 (55.8%)	0.04
	NVD	18 (81.8%)	4 (18.2%)	
Gestational age	Late Preterm	6 (33.3%)	12 (66.7%)	0.03
	Term	35 (69.4%)	21 (30.6%)	
Birth weight	LBW	7 (28.0%)	18(72.0%)	0.001
	NBW	34 (69.4%)	15 (30.6%)	
Gender	Female	17 (63.0%)	10 (37.0%)	0.322
	Male	24 (51.1%)	23 (48.9%)	
Maternal comorbidities	Hypothyroid	0	1 (100%)	0.269
	GDM	1 (50%)	1 (50%)	
	Pre-eclampsia	0	2 (100%)	
	No comorbidities	40 (58%)	29 (42%)	
Downe’s score	1-4	36 (63.2%)	21 (36.8%)	0.025
	5-6	5 (29.4%)	12 (70.6%)	

## Discussion

In our study, the incidence of TTN was 16 per 1000 live births. TTN accounted for 5.3% of all the cases admitted to our NICU. It was a leading cause of respiratory distress accounting for 39% of respiratory distress in term and late preterm infants. This was closer to reported incidences of TTN in India: 13 per 1000 live births in Northern India by Thomas et al. (1981) and 28 per 1000 live births in Southern India by Kumar and Bhat (1996) [6,7]. A review of unpublished five years (2010-2016) of data from All India Institutes of Medical Sciences (AIIMS), a public sector apex referral center in India’s capital, reported the incidence of TTN to be as high as 46.6 per 1000 live births [8]. The incidence of TTN in India seems to be higher compared to high-resource countries. Most of these reports on incidence from India are from tertiary care referral centers, which see a large volume of referrals of high-risk pregnancies, thus having a higher number of LSCS deliveries. Also, the rates of cesarean sections have been on a rising trend in India. Eighteen percent of deliveries in urban India and 5% of deliveries in rural India were via LSCS [9], which might have led to an increased incidence of TTN. TTN was more common in the male patients (63.5%) compared to the female (36.5%) patients in this study. This was similar to findings by Kasap et al. and Tutdibi et al., both of whom reported male sex as a risk factor for TTN. This risk is probably due to the difference in lung growth and maturation in both sexes [10,11]. Dani et al. in a nationwide Italian study reported male sex to be a risk factor for both TTN and respiratory distress syndrome (RDS) [12]. LSCS has been an established risk factor for TTN. Liem et al. and Tutdibi et al. found an increase of TTN by two- to three-fold in the neonates delivered by elective cesarean section than those born vaginally [11,13]. Seventy percent of our patients were born via LSCS. Infants delivered through elective cesarean sections often are deprived of the labor-related physiological stress response pattern at birth and consequently experience failure of postnatal respiratory transition. Delaying elective cesarean sections until after 39 weeks of gestation has been shown to reduce respiratory morbidity in newborns. The American College of Obstetrics and Gynecology (2019) recommends avoiding vaginal or cesarean deliveries at less than 39 weeks gestation unless indicated medically [14]. We found that newborns delivered via LSCS had a longer duration of tachypnea than those born vaginally. The mean duration of tachypnea in the LSCS group was 32.4 hours, and in the vaginal delivery group was 26.2 hours. Tachypnea had resolved within 24 hours in 81.8% of patients in the vaginal delivery group, while in the LSCS group only 44.2% had shown resolution of tachypnea by 24 hours. The majority (66.2%) of the infants in our study were normal birth weight (2.5 Kg-4.0 Kg). This was in disagreement with Liem et al. and Tutdibi et al., who had reported a significant relationship between TTN and low birth weight [13,11]. Dani et al. in their study involving 63,537 newborns investigated the risk factors for RDS and TTN. He reported low birth weight to be a risk factor for both [12].

Kweon et al. had suggested that the duration of tachypnea lasted longer in low birth weight infants [15]. Our findings were similar: the tachypnea had resolved by 24 hours in most NBW infants (mean: 26.3 hrs.) in our study, while LBW infants needed a longer duration (mean: 38.9 hrs.) of hospital stay. The association between parity and TTN is not established. Takaya et al. had reported that nulliparity was a risk factor for the development of TTN [16]. No association between parity and TTN was found in our study. The number of multigravidas (n=36) and primigravida (n=38) were approximately equal in our study. Also, parity did not dictate any difference in the severity of distress, outcome, or duration of distress. Prematurity has been associated as a risk factor for TTN [10-12]. However, in our study, three-fourths (75.7%) of the neonates were term infants. The term infants showed earlier resolution of tachypnea, with two-thirds of them recovering within the first 24 hours. Bivariate analysis of gestational age and duration of tachypnea showed a significant negative correlation (p<0.01, Correlation Coefficient= -0.426). Gestational diabetes mellitus and maternal asthma are well-established risk factors for the development of TTN [10,17,18]. Badran et al. (2012) found that maternal hypertension, diabetes mellitus, and the absence of labor were independent risk factors for respiratory morbidity in neonates [17]. In a large nationwide Swedish study (1991-93), rates of asphyxia and transient tachypnea were two to three times higher in patients with gestational diabetes mellitus compared to the normal population [18]. In our study, there were two cases of preeclampsia, one of hypothyroidism, and two of gestational diabetes mellitus. All of them had delivered via LSCS. The duration of distress in all of them lasted more than 24 hours. There was no significant difference between the mean duration among the groups. None of these newborns needed CPAP; oxygen supplementation with nasal prongs was sufficient.

The strength of this study includes a large sample size, and there was a thorough and meticulous evaluation of all cases of neonatal respiratory distress and their follow-up till discharge.

A limitation of this study is the low number of patients with maternal comorbidities, so their impact on outcomes of TTN is not reflected in the findings. Also, being a tertiary care center that handles high-risk deliveries majority of which undergo LSCS, the incidence thus is high. This incidence may not reflect the true incidence in the community.

## Conclusions

TTN is the most common cause of respiratory distress among late preterm and term newborns. Oxygen supplementation and supportive therapy were mainstays of management, and no complications were seen. Delivery by LSCS and male sex are major risk factors for the development of TTN. Our study demonstrates that most patients will improve within the first 48 hours. Higher Downes' scores on presentation, cesarean delivery, low birth weight, and prematurity are predictors of a longer duration of hospital stay. Larger study samples are required to establish the complications of TTN (PPHN, prolonged tachypnea, air leaks) and factors that might predict them.
